# Association between Antenatal Vaginal Bleeding and Adverse Perinatal Outcomes in Placenta Accreta Spectrum

**DOI:** 10.3390/medicina60040677

**Published:** 2024-04-22

**Authors:** J. Connor Mulhall, Kayla E. Ireland, John J. Byrne, Patrick S. Ramsey, Georgia A. McCann, Jessian L. Munoz

**Affiliations:** 1 Department of Obstetrics and Gynecology, Division of Maternal Fetal Medicine, Division of Fetal Intervention, Baylor College of Medicine, Texas Children’s Hospital, Houston, TX 77030, USA; joseph.mulhall@bcm.edu; 2 Department of Obstetrics & Gynecology, University of Texas Health Sciences Center at San Antonio, San Antonio, TX 78229, USA; ireland@uthscsa.edu (K.E.I.); byrnej@uthscsa.edu (J.J.B.); ramseyp@uthscsa.edu (P.S.R.); mccanng@uthscsa.edu (G.A.M.)

**Keywords:** placenta accreta spectrum, antepartum hemorrhage, antenatal vaginal bleeding, ultrasound, cesarean hysterectomy

## Abstract

*Background and Objectives*: Placenta accreta spectrum (PAS) disorders are placental conditions associated with significant maternal morbidity and mortality. While antenatal vaginal bleeding in the setting of PAS is common, the implications of this on overall outcomes remain unknown. Our primary objective was to identify the implications of antenatal vaginal bleeding in the setting of suspected PAS on both maternal and fetal outcomes. *Materials and Methods*: We performed a case-control study of patients referred to our PAS center of excellence delivered by cesarean hysterectomy from 2012 to 2022. Subsequently, antenatal vaginal bleeding episodes were quantified, and components of maternal morbidity were assessed. A maternal composite of surgical morbidity was utilized, comprised of blood loss ≥ 2 L, transfusion ≥ 4 units of blood, intensive care unit (ICU) admission, and post-operative length of stay ≥ 4 days. *Results*: During the time period, 135 cases of confirmed PAS were managed by cesarean hysterectomy. A total of 61/135 (45.2%) had at least one episode of bleeding antenatally, and 36 (59%) of these had two or more bleeding episodes. Increasing episodes of antenatal vaginal bleeding were associated with emergent delivery (*p* < 0.01), delivery at an earlier gestational age (35 vs. 34 vs. 33 weeks, *p* < 0.01), and increased composite maternal morbidity (76, 84, and 94%, *p* = 0.03). *Conclusions*: Antenatal vaginal bleeding in the setting of PAS is associated with increased emergent deliveries, earlier gestational ages, and maternal composite morbidity. This important antenatal event may aid in not only counseling patients but also in the coordination of multidisciplinary teams caring for these complex patients.

## 1. Introduction

The placenta accreta spectrum (PAS) refers to the continuum of abnormal implantation and invasion of a portion or the entirety of the placenta into the myometrial layer of the uterus during pregnancy [1]. The exact underlying pathophysiology of PAS remains unknown [2]. Formerly known as morbidly adherent placenta, PAS was also classically stratified by the depth that the placenta invaded through the myometrium (accreta, increta, and percreta). Those three subtypes can be determined by pathological or clinical assessment of invasion, with accreta referring to placental adherence to the myometrium, increta if the myometrium is invaded, and percreta when the depth of invasion extends to the outer uterine serosa [3,4]. In 2019, the development of a more detailed system was developed by the International Federation of Gynecology and Obstetrics (FIGO) [5]. Subsequently, an expert panel developed a pathology-based categorization for cases of PAS [6].

The current detection of PAS is dependent on clinical suspicion and consideration of patient-specific historical risk factors. A primary risk factor for PAS is a history of cesarean delivery in a prior pregnancy [7]. A direct relationship is observed between the number of prior cesarean deliveries and PAS risk. As global cesarean section rates continue to increase, so does the incidence of PAS [7,8]. This is displayed by the prevalence of PAS in the United States, which rose more than 6-fold from 1982 to 2002 to a rate of 0.2% [8].

Pregnancies complicated by PAS carry risks for significant maternal adverse outcomes. The atypical placental attachment prohibits the normal separation and expulsion of the placenta, thus inhibiting subsequent uterine involution. This leads to increased rates of postpartum hemorrhage, which can be life-threatening and require a blood transfusion. Additionally, the classic management of PAS is surgical via hysterectomy at the time of cesarean delivery [9]. Due to the depth of placental invasion and obliteration of standard surgical planes, there is an increased risk for intraoperative organ injury, particularly of the genitourinary (GU) system [9,10]. Adverse neonatal outcomes are primarily driven by the degree of prematurity at the time of delivery and exposure to general anesthesia [11,12].

Ultrasonography remains the gold standard for the identification of findings concerning PAS during pregnancy [10]. The strongest risk factor for PAS is the presence of placenta previa, a term describing the placenta covering all or part of the internal cervical os [7]. Additional findings that can raise suspicion for PAS include placental lacunae, thinning of the myometrial layer, loss of the normal hypoechogenicity between the placenta and myometrium (hereby referred to as retroplacental clear space), and increased vascularity on Doppler imaging. Magnetic resonance imaging (MRI) may be a useful adjunct if the diagnosis is not clear and to assist with surgical planning, although it may have poor predictive value overall [13]. Early diagnosis of PAS is imperative to allow for delivery to occur at a high-resource medical center [14]. These cases require a multi-disciplinary approach, often including maternal-fetal medicine, neonatology, anesthesiology, urology, and gynecologic oncology [15].

Given the highly coordinated nature of these cases, it is recommended that delivery occur prior to the onset of labor to allow for adequate preparation by all appropriate teams [16]. While later gestational age at delivery can improve neonatal outcomes, delivery after 34 weeks increases the rate of antepartum hemorrhage, which can be life-threatening for both in the maternal-fetal dyad [17]. Antepartum hemorrhage, hereby referred to as antenatal vaginal bleeding, significantly increases the risk of an unscheduled delivery in patients with PAS, and this risk increases further if preterm prelabor rupture of membranes (PPROM) also occurs [16]. An increased number of antenatal vaginal bleeding episodes has also been associated with an earlier gestational age at the time of delivery.

Antenatal vaginal bleeding as early as the first trimester of pregnancy is associated with poor obstetric outcomes, including preterm birth, miscarriage, and placental abruption [18]. Vaginal bleeding during the latter half of pregnancy has been associated with adverse neonatal outcomes in patients without placenta previa or PAS. Yet, data from the ADoPAD study group did not identify any antenatal sonographic findings associated with emergent delivery or maternal morbidity in cases of placenta previa with or without PAS [19]. However, episodes of bleeding were not considered, and thus there is a lack of data regarding both maternal and neonatal outcomes from patients with PAS who experience antenatal vaginal bleeding during pregnancy and how to counsel these patients.

In this study, we aim to compare perinatal outcomes between patients with PAS based on the occurrence of antenatal vaginal bleeding and to further stratify these outcomes based on the number of bleeding events that occurred. Our primary outcome was a previously reported composite of maternal morbidity in patients with PAS undergoing hysterectomy [20].

## 2. Materials and Methods

### 2.1. Study Design

We performed a case-control study and presented it to the University Health Systems center for PAS management between 2012 and 2022. Institutional review board (IRB) approval was obtained from the University of Texas Health San Antonio and University Hospital System prior to obtaining patient information from electronic medical records. Due to the nature of the retrospective study, patient consent was not required. Inclusion criteria were: viable pregnancy, maternal age between 18 and 55, antenatal suspicion for PAS (based on historical risk factors, ultrasound, or MRI findings), and histopathological confirmation/characterization of PAS by a board-certified pathologist. Patients were excluded if they delivered at another institution after PAS consultation, had a gestational age < 20 weeks, or had incomplete medical records.

Our center for PAS care provides evidence-based multidisciplinary care, including consultation with several specialists/subspecialists, including maternal-fetal medicine, urology, gynecologic oncology, obstetric anesthesia, transfusion medicine, interventional radiology, and trauma surgery.

### 2.2. Surgical Approach

Current approaches to PAS include planned cesarean hysterectomy, cesarean delivery with the placenta left in situ, or delivery with uterine reconstruction/preservation. Our center did not practice conservative or reconstructive management of PAS at the time of this study, and thus, all patients were managed by planned cesarean hysterectomy. Our program utilizes an antenatal stratification approach to the staffing of cases and the active teaching of trainees, both residents and fellows [21]. In cases of suspected placenta accreta, a hysterectomy was performed by a staff gynecologist from the interdisciplinary team. In cases of suspected placenta increta/percreta, a staff gynecologic oncologist performed the hysterectomy. Ureteral stents were planned for all cases when they were clinically feasible and safe. The selection of patient anesthesia approach was determined by the staff anesthesiologist in consideration of the patient’s desires and clinical acuity. In cases of suspected placenta percreta, after ureteral stent placement, vascular access was obtained by the interventional radiologist for the use of uterine artery embolization (UAE). Thus, after delivery of the neonate, the hysterotomy was closed and UAE was performed prior to the hysterectomy. This approach has been reported by our team [22].

### 2.3. Study Outcomes

Our primary outcome was a previously reported composite of maternal morbidity, which incorporates aspects of both intraoperative and postoperative complications [20]. In addition, aspects of maternal baseline conditions, ultrasound assessment, pregnancy complications, and operative characteristics were obtained from electronic medical records.

Antenatal vaginal bleeding was determined by individual events of bleeding beyond spotting, as verified by trained medical staff. Unless bleeding was present on admission or evaluation in the hospital, at-home bleeding reported by the patient was not counted, as this could not be verified to be greater than minimal/spotting. Due to the spontaneous nature of antenatal vaginal bleeding, the overall volumes of bleeding were not quantified, only episodes of bleeding.

### 2.4. Statistical Analysis and Data Storage

Research Electronic Data Capture (REDCap) software hosted by the University of Texas Health San Antonio was used for data collection and storage. REDCap (Research Electronic Data Capture, Vanderbilt University, Nashville, TN, USA) is a secure, web-based application designed to support data capture for research studies. Data were extracted from the electronic medical record and manually entered into REDCap by the investigators. This study followed the Strengthening the Reporting of Observational Studies in Epidemiology (STROBE) reporting guideline. Normal distribution was determined by a Shapiro–Wilk test greater than 0.05. Pearson’s chi-square (χ^2^), Fisher’s exact tests, the Mann–Whitney U test, and T-tests were applied when appropriate. Categorical factors were summarized using frequencies and percentages, while continuous measure summaries used means ± SD or median and range as appropriate. Kaplan-Meier curves were developed for all three groups with respect to pregnancy time intervals of delivery to assess for the gestational age of delivery for each group. *p*-values < 0.05 were considered significant for a two-tailed analysis. Statistical analysis was performed using Graphpad software (version 10.0.2).

## 3. Results

During the study period, 135 patients had PAS confirmed by pathology. Among this cohort, 78 (57.7%) had no antenatal vaginal bleeding during pregnancy, 25 (18.5%) had one episode of bleeding, and 36 (26.7%) patients had two or more episodes of antenatal vaginal bleeding. The mean gestational age for those with one bleed was 27 ± 7.4 weeks. For those with two episodes of bleeding or more, the mean gestational ages were: 24 ± 8.9 weeks (first), 29 ± 5 weeks (second), and 30 ± 7.1 weeks (third). The latency period between the first and second bleeding episodes was 4.8 ± 6.7 weeks, and then 3.1 ± 3.7 weeks between the second and third episodes. Table 1 details the demographic variables of the cohort categorized by the number of episodes of antenatal vaginal bleeding. Clinical characteristics between the three groups were similar, with two exceptions. Patients with more than two episodes of bleeding were more likely to be current smokers and also were more likely to require emergent delivery.

Ultrasonographic findings for all patients were assessed. Patients who experienced antenatal vaginal bleeding were more likely to have placental lacunae identified on ultrasound. Neither the presence nor recurrence of antenatal vaginal bleeding during pregnancy correlated with other ultrasonographic findings concerning PAS. As detailed in Table 2, there was no difference in the antenatal diagnosis of PAS or of any of the three PAS subtypes on the basis of antenatal vaginal bleeding.

Table 3 summarizes the antepartum complications that occurred within the cohort. Overall, this population had a high rate of antepartum admissions, though patients who had multiple episodes of antenatal vaginal bleeding were significantly more likely to be hospitalized during pregnancy. Additionally, this group had a significantly longer hospital length of stay (LOS) than those who had one or no episodes of bleeding. There was no difference in the rates of PPROM, preterm labor, fetal growth restriction, hypertensive disorders of pregnancy, or gestational diabetes between groups.

In regards to delivery and operative outcomes (Table 4), patients who experienced antenatal vaginal bleeding tended to deliver at earlier gestational ages, and those who had multiple episodes of bleeding were delivered earlier than those with only one episode. Estimated blood loss (EBL) during delivery was significantly greater for the patients who experienced multiple episodes of antenatal vaginal bleeding during pregnancy. Additionally, this group had significantly longer postoperative LOS. There was no difference in operative time, use of general anesthesia, performance of uterine artery embolization (UAE), or intraoperative GU injury.

**Figure 1:**
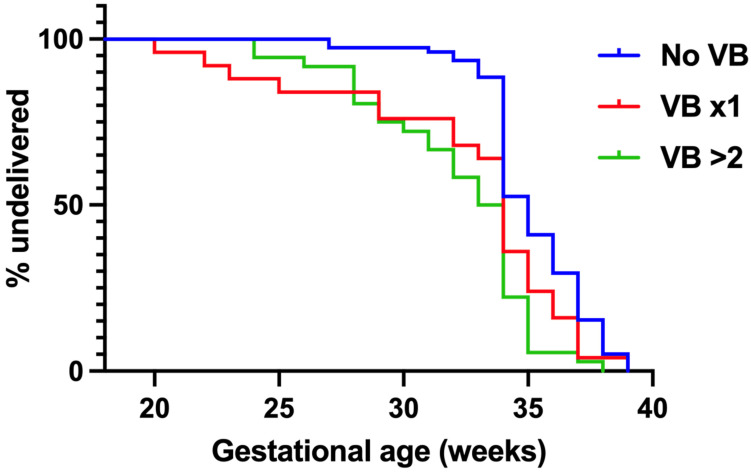
Kaplan-Meier survival curve, defined as gestational age at delivery in weeks.

A Kaplan-Meier curve was obtained for further assessment of gestational age and antenatal vaginal bleeding (Figure 1). When compared to no episodes of antenatal vaginal bleeding (blue line), 1 (red line) and ≥2 (green line) episodes of antenatal vaginal bleeding were associated with earlier gestational ages of delivery, particularly in the second trimester and early third trimester.

The composite maternal morbidity is presented in Table 5. In the total cohort, 115 patients (85.2%) experienced at least one adverse maternal outcome, and the likelihood of morbidity significantly increased if the patient had an episode of antenatal vaginal bleeding, and again so if they experienced multiple bleeding events. Among the individual components of the composite, those with multiple occurrences of antenatal vaginal bleeding in pregnancy were more likely to require transfusion of four or more units of blood, be admitted to the ICU, and have a postoperative length of stay greater than four days. There was no significant difference in the rate of EBL greater than 2 L between the groups.

## 4. Discussion

Our data show the importance of antenatal bleeding episodes in counseling patients and the potential increased morbidity associated with these episodes. At present, maternal morbidity remains high, and the incidence of placenta accreta spectrum continues to increase as a result of the increased rate of cesarean sections, with most recent data estimating almost 1 in 500 pregnancies complicated by this condition [23]. As such, there have been efforts to raise awareness of this condition amongst both medical professionals and the general public [24]. Advances in ultrasound and MRI technology have improved the detection of markers concerning PAS, yet several limitations exist in the overall accuracy of antenatal imaging. Medical professionals should have a high index of suspicion for PAS based on historical risk factors and in the context of ultrasonographic findings. PAS cases detected at the time of delivery have higher rates of complications than those diagnosed antenatally and do not allow for optimal team-based management.

There have been recent endeavors to develop centers of excellence for the management of PAS, the goal of which is to emphasize the importance of a multidisciplinary care team with expertise and experience with the condition [25]. These centers have shown improved outcomes for patients who deliver at a medical center with experienced team members. Yet, the greatest limitation remains: these centers are dependent on the antenatal detection and prompt referral of PAS cases. Novel operative techniques, advanced surgical technology, and the use of adjunctive treatments have all moved the needle in terms of improved patient safety for those with PAS.

Despite these advancements, pregnancies complicated by PAS continue have significant adverse outcomes. In our study population, the majority of patients experienced at least one complication included in the maternal morbidity composite. Patients with multiple bleeding episodes were significantly more likely to experience an adverse outcome, and this significance was present for all individual components of the composite with the exception of EBL ≥ 2 L. Yet, the overall blood loss is high across groups. While there were more emergent deliveries in the group who experienced repeat occurrences of antenatal vaginal bleeding, prior studies have not shown an association between emergent delivery and adverse maternal outcomes [26,27].

Multiple scoring systems and prediction models have been developed in an attempt to stratify patients’ risk for adverse events. The Placenta Accreta Index (PAI) correlates patients’ prior cesarean section history, as well as ultrasound markers to predict the likelihood of PAS as a potential proxy for risk of morbidity [28]. The Placenta Accreta Risk-Antepartum (PAR-A) and Placenta Accreta Risk-Peripartum (PAR-P) scores are machine-learning-based tools that use several patient demographics, medical histories, and imaging data to predict morbidity in patients with PAS [27]. While these tools can aid in perioperative risk assessment, they each have their limitations in terms of specificity and generalizability. None of these tools assess for antenatal vaginal bleeding. We did not employ these assessment scores in our patient cohort, and we did not find a strong association between the ultrasound findings of PAS and the occurrence of antenatal vaginal bleeding, with the exception of the presence of placental lacunae. Given the nature of this study, we are unable to conclude whether the presence of placental lacunae predisposes patients to antenatal vaginal bleeding during pregnancy. In the setting of placenta previa, antenatal vaginal bleeding is associated with poor obstetric outcomes. Ultrasound evaluation of bleeding in this context is greater in those with a short cervix [29]. In addition, prior studies have also shown placental lacunae on ultrasound do correlate with antenatal bleeding in the setting of placenta previa (without PAS) [30].

This study did not consider vaginal spotting or unconfirmed out-of-hospital bleeding for uniformity of evaluation; thus, the impact of minor bleeding episodes cannot be inferred from our data. In addition, spontaneous bleeding did not allow for the quantification of bleeding during these episodes. Yet, this approach does allow for a practical interpretation of antenatal vaginal bleeding since most bleeding episodes are not precisely quantified.

The data presented in our study show an association between antenatal vaginal bleeding in pregnancy and an earlier gestational age of delivery. This is consistent with prior data showing a similar conclusion. This study is novel in that not only does it suggest adverse neonatal outcomes related to prematurity if delivered earlier due to antenatal vaginal bleeding, but it also shows an association with worsened maternal outcomes if recurrent antenatal vaginal bleeding episodes occur. In 2010, Robinson and Grobman proposed 34 weeks as the optimal gestational age of delivery for pregnancies complicated by PAS in most cases [14]. Their analysis also showed that the probability of antenatal vaginal bleeding requiring delivery increased with increasing gestational age. While our study design inhibits the ability to draw conclusions regarding delivery timing, our data do suggest that delaying delivery and increasing the risk for antenatal vaginal bleeding could increase the risk for adverse maternal outcomes as well.

Here we present novel data on maternal outcomes from pregnancies complicated by antenatal vaginal bleeding during pregnancies affected by PAS, as these have not been considered previously. The limitations of our study include its retrospective nature, and thus, our inability to prove the causality of our findings. Also, patients in this cohort come from a single institution, which may limit its generalizability. In addition, as previously described, our center has a series of protocols for PAS management that may vary from those of other institutions. Lastly, additional prenatal and pathologic categories of PAS have been reported; these were described in recent years, and our student extends to years preceding these systems, thus they could not be included in our analysis.

The strengths of this study include its relatively large sample size for a rare obstetric condition. Also, all cases were managed by the same interdisciplinary team which employed protocolized approaches to PAS care, thus reducing variability among case management. The use and practice of protocols also allowed for implementation in emergent situations. As previously noted, our center does not employ “conservative” approaches to PAS care and thus our outcomes may vary from centers that do. Recently, a randomized trial showed a reduction in blood transfusion and associated morbidity when managed conservatively with uterine reconstruction [31]. Additionally, we only included cases where PAS was confirmed by pathologic diagnosis, not only on antenatal ultrasound findings. This approach was optimal given the variability in antenatal detection by imaging. To date, no validate biomarkers are in clinical practice and thus were not explored in thus study, but present a clinical area in need of objective assessment. Prospective studies from multiple centers are necessary to determine the validity and generalizability of the findings we present here.

Lastly, patient counseling remains a cornerstone of PAS care. As such, discovering clinically relevant risk factors for predicting maternal morbidity allows for evidence-based counseling and the establishment of expectations. These approaches have been used previously to counsel on potential needs for blood transfusion in the setting of PAS. Interestingly, data predicting blood transfusion did identify antenatal vaginal bleeding ≥ 2 episodes as an independent risk factor in the setting of PAS [32].

## 5. Conclusions

The management of pregnancies complicated by PAS requires a highly specialized and multidisciplinary care team. Despite ongoing advancements in the understanding of the disease and how best to care for these patients, ongoing research is required to determine best practices. Our study suggests that there is an association between antenatal vaginal bleeding during pregnancy and adverse maternal outcomes. Additionally, we show that patients who experience antenatal vaginal bleeding may deliver at earlier gestational ages than those without bleeding. We hope this work helps spur future prospective studies to both corroborate our findings and improve outcomes for patients with PAS.

## Figures and Tables

**Table 1:** Study group demographics.

	No AVB	AVB x1	AVB ≥ 2	
	(n = 78)	(n = 25)	(n = 36)	*p*-Value
Age (years)	31.2 ± 5.6	30.9 ± 5.8	31.8 ± 4.8	0.82
BMI (kg/m^2^)	33.3 ± 5.8	32.5 ± 6.5	32.3 ± 6.1	0.61
Gravidity	5 [4,6]	4 [3,7]	5 [4,6]	0.69
Parity	3 [2,4]	2 [2,3]	3 [2,4]	0.77
Prior CD	73 (94)	24 (96)	33 (92)	0.66
Number of prior CD	3 [2,4]	2 [2,3]	3 [2,3]	0.34
Pregestational diabetes	8 (10)	3 (12)	0 (0)	0.08
Chronic hypertension	7 (9)	5 (20)	2 (6)	0.20
Anemia	29 (37)	10 (40)	11 (31)	0.74
Active smoking	2 (3)	2 (8)	5 (14)	**0.04**
Emergent delivery	6 (8)	10 (40)	24 (67)	**<0.01**
Public insurance	61 (78)	21 (58)	25 (69)	0.42

Values presented as Mean ± SD, Median [P25, P75], or N (column %) BMI = body mass index, CD = cesarean delivery, AVB = antenatal vaginal bleeding Bold *p*-value < 0.05.

**Table 2:** Ultrasound findings.

	No AVB	AVB x1	AVB ≥ 2	
	(n = 78)	(n = 25)	(n = 36)	*p*-Value
Antenatal diagnosis				
No evidence of PAS	32 (41)	6 (24)	10 (28)	0.21
Accreta	28 (36)	12 (48)	17 (47)	0.40
Increta	1 (1)	1 (4)	1 (3)	0.41
Percreta	17 (22)	6 (24)	8 (22)	0.96
Placental lacunae	31 (40)	17 (68)	22 (61)	**0.02**
Loss of RPCS	22 (28)	10 (40)	14 (39)	0.37
Hypervascularity	26 (33)	8 (32)	15 (42)	0.65
Myometrial thinning (<2 mm)	21 (27)	10 (40)	11 (31)	0.47
Feeder vessels	6 (8)	1 (4)	1 (3)	0.70

Values presented as N (column %) Retroplacental clear space = RPCS Bold *p*-value < 0.05.

**Table 3:** Antepartum complications.

	No AVB	AVB x1	AVB ≥ 2	
	(n = 78)	(n = 25)	(n = 36)	*p*-Value
Antepartum admission	49 (63)	16 (64)	31 (86)	0.82
Antepartum LOS	1 [0,2]	1 [0,6]	7 [7,25]	0.61
PPROM	3 (4)	0 (0)	5 [4,6]	0.69
Preterm labor	4 (5)	1 (4)	3 [2,4]	0.77
Fetal growth restriction	3 (4)	0 (0)	33 (92)	0.66
Gestational hypertension	3 (4)	0 (0)	3 [2,3]	0.34
Pre-eclampsia without SF	1 (1)	0 (0)	0 (0)	0.08
Pre-eclampsia with SF	3 (4)	0 (0)	2 (6)	0.20
Gestational diabetes	11 (14)	3 (12)	11 (31)	0.74

Values presented as Median [P25, P75] or N (column %) LOS = length of stay, PPROM = preterm prelabor rupture of membranes, SF = severe features.

**Table 4:** Intra- and post-operative complications.

	No AVB	AVB x1	AVB ≥ 2	
	(n = 78)	(n = 25)	(n = 36)	*p*-Value
Gestational age (weeks)	35 [34,37]	34 [31,36]	33 [29,34]	**<0.01**
Admission Hgb	10.9 ± 1.4	10.7 ± 1.4	11.0 ± 1.2	0.70
Operative time (min)	191 [141,323]	198 [164,259]	223 [154,347]	0.55
EBL (mL)	2321 [1500,4000]	2500 [2000,4000]	4000 [2000,6500]	**<0.01**
General anesthesia	36 (46)	16 (64)	20 (56)	0.27
UAE performed	15 (19)	4 (16)	7 (19)	0.99
GU injury	20 (26)	4 (16)	13 (36)	0.22
ICU LOS	0 [0,1]	0 [0,1]	1 [0,2]	0.90
Postoperative LOS (days)	3 [3,5]	3 [3,5]	4 [3,5]	**0.04**

Values presented as Mean ± SD, Median [P25, P75], or N (column %) Hgb = hemoglobin, EBL = estimated blood loss; UAE = uterine artery embolization, GU = genitourinary, ICU = intensive care unit, LOS = length of stay Bold *p*-value < 0.05.

**Table 5:** Composite maternal morbidity.

	No AVB	AVB x1	AVB ≥ 2	
	(n = 78)	(n = 25)	(n = 36)	*p*-Value
Maternal morbidity composite	59 (76)	22 (84)	34 (94)	**0.03**
EBL ≥ 2 L	55 (71)	19 (76)	32 (89)	0.1
Transfusion ≥ 4 units	35 (45)	15 (60)	25 (69)	**0.04**
ICU admission	39 (37)	8 (32)	23 (64)	**0.01**
LOS ≥ 4 days	31 (40)	12 (48)	26 (72)	**<0.01**

Values presented as N (column %) Bold *p*-value < 0.05.

## Data Availability

The data presented in this study are available on request from the corresponding author due to patient privacy.

## References

[1] Cahill A.G., Beigi R., Heine R.P., Silver R.M., Wax J.R., American College of Obstetricians and Gynecologists (2018). Placenta accreta spectrum. Am. J. Obstet. Gynecol..

[2] Einerson B.D., Kennedy A., Silver R.M., Branch D.W., Comstock J., Woodward P.J. (2023). Ultrasonography of the explanted uterus in placenta accreta spectrum: Correlation with intraoperative findings and gross pathology. Obstet. Gynecol..

[3] Einerson B.D., Comstock J., Silver R.M., Branch D.W., Woodward P.J., Kennedy A. (2020). Placenta accreta spectrum disorder: Uterine dehiscence, not placental invasion. Obstet. Gynecol..

[4] Silver R.M., Barbour K.D. (2015). Placenta accreta spectrum: Accreta, increta, and percreta. Obstet. Gynecol. Clin. N. Am..

[5] Cali G., Forlani F., Lees C., Timor-Tritsch I., Palacios-Jaraquemada J., Dall’asta A., Bhide A., Flacco M.E., Manzoli L., Labate F. (2019). Prenatal ultrasound staging system for placenta accreta spectrum disorders. Ultrasound Obstet. Gynecol..

[6] Hecht J.L., Baergen R., Ernst L.M., Katzman P.J., Jacques S.M., Jauniaux E., Khong T.Y., Metlay L.A., Poder L., Qureshi F. (2020). Classification and reporting guidelines for the pathology diagnosis of placenta accreta spectrum (PAS) disorders: Recommendations from an expert panel. Mod. Pathol..

[7] Wu S., Kocherginsky M., Hibbard J.U. (2005). Abnormal placentation: Twenty-year analysis. Am. J. Obstet. Gynecol..

[8] Silver R.M., Branch D.W. (2018). Placenta accreta spectrum. N. Engl. J. Med..

[9] Tam K.B.T., Dozier J., Martin J.N. (2012). Approaches to reduce urinary tract injury during management of placenta accreta, increta, and percreta: A systematic review. J. Matern. Fetal. Neonatal Med..

[10] D’Antonio F., Iacovella C., Bhide A. (2013). Prenatal identification of invasive placentation using ultrasound: Systematic review and meta-analysis. Ultrasound Obstet. Gynecol..

[11] Munoz J.L., Kimura A.M., Julia J., Tunnell C., Hernandez B., Curbelo J., Ramsey P.S., Ireland K.E. (2022). Impact of placenta accreta spectrum (PAS) pathology on neonatal respiratory outcomes in cesarean hysterectomies. J. Matern. Fetal. Neonatal Med..

[12] Toussia-Cohen S., Castel E., Friedrich L., Mor N., Ohayon A., Levin G., Meyer R. (2024). Neonatal outcomes in pregnancies complicated by placenta accreta- a matched cohort study. Arch. Gynecol. Obstet..

[13] Einerson B.D., Rodriguez C.E., Silver R.M., Donnelly M.A., Kennedy A.M., Woodward P.J. (2021). Accuracy and interobserver reliability of magnetic resonance imaging for placenta accreta spectrum disorders. Am. J. Perinatol..

[14] Robinson B.K., Grobman W.A.M. (2010). Effectiveness of timing strategies for delivery of individuals with placenta previa and accreta. Obstet. Gynecol..

[15] Fitzgerald G.D., Newton J., Atasi L., Buniak C.M., Burgos-Luna J.M., Burnett B.A., Carver A.R., Cheng C., Conyers S., Davitt C. (2024). Placenta accreta spectrum care infrastructure: An evidence-based review of needed resources supporting placenta accreta spectrum care. Am. J. Obstet. Gynecol. MFM.

[16] Bowman Z.S., Manuck T.A., Eller A.G., Simons M., Silver R.M. (2014). Risk factors for unscheduled delivery in patients with placenta accreta. Am. J. Obstet. Gynecol..

[17] Harlev A., Levy A., Zaulan Y., Koifman A., Mazor M., Wiznitzer A., Faizayev E., Sheiner E. (2008). Idiopathic bleeding during the second half of pregnancy as a risk factor for adverse perinatal outcome. J. Matern. Fetal. Neonatal Med..

[18] Karimi A., Sayehmiri K., Vaismoradi M., Dianatinasab M., Daliri S. (2024). Vaginal bleeding in pregnancy and adverse clinical outcomes: A systematic review and meta-analysis. J. Obstet. Gynaecol..

[19] Group A.D.S. (2024). Determinants of emergency cesarean delivery in pregnancies complicated by placenta previa with or without placenta accreta spectrum disorder: Analysis of adopad cohort. Ultrasound Obstet. Gynecol..

[20] Munoz J.L., Ramsey P.S., Byrne J.J. (2023). Risk of severe maternal morbidity in patients with placenta accreta spectrum disorders referred from rural communities to a regional placenta accreta spectrum center. Am. J. Perinatol..

[21] Munoz J.L., Blankenship L.M., Ramsey P.S., McCann G.A. (2022). Importance of the gynecologic oncologist in management of cesarean hysterectomy for placenta accreta spectrum (pas). Gynecol. Oncol..

[22] Munoz J.L., Blankenship L.M., Ramsey P.S., McCann G.A. (2023). Implementation and outcomes of a uterine artery embolization and tranexamic acid protocol for placenta accreta spectrum. Am. J. Obstet. Gynecol..

[23] Silver R.M., Fox K.A., Barton J.R., Abuhamad A.Z., Simhan H., Huls C.K., Belfort M.A., Wright J.D. (2015). Center of excellence for placenta accreta. Am. J. Obstet. Gynecol..

[24] Castillo J., Zhu K., Gray L., Sachse S., Berra A., Belfort M.A., Aalipour S., Aagaard K.M., Shamshirsaz A.A. (2023). Youtube as a source of patient information regarding placenta accreta spectrum. Am. J. Perinatol..

[25] Zuckerwise L.C., Craig A.M., Newton J., Zhao S., Bennett K.A., Crispens M.A. (2020). Outcomes following a clinical algorithm allowing for delayed hysterectomy in the management of severe placenta accreta spectrum. Am. J. Obstet. Gynecol..

[26] Shazly S.A., Hortu I., Shih J.-C., Melekoglu R., Fan S., Ahmed F.U.A., Karaman E., Fatkullin I., Pinto P.V., Irianti S. (2022). Prediction of clinical outcomes in women with placenta accreta spectrum using machine learning models: An international multicenter study. J. Matern. Neonatal Med..

[27] Shazly S.A., Anan M.A., Makukhina T.B., Melekoglu R., Ahmed F.U.A., Pinto P.V., Takahashi H., Ahmed N.B., Sayed E.G., Elassall G.M. (2022). Placenta accreta risk—Antepartum score in predicting clinical outcomes of placenta accreta spectrum: A multicenter validation study. Int. J. Gynecol. Obstet..

[28] Happe S.K., Yule C.S., Spong C.Y., Wells C.E., Dashe J.S., Moschos E., Rac M.W., McIntire D.D., Twickler D.M. (2021). Predicting placenta accreta spectrum: Validation of the placenta accreta index. J. Ultrasound Med..

[29] Smith D.D., Adesomo A.A., Gonzalez-Brown V.M., Russo J., Shellhaas C., Costanstine M.M., Frey H.A. (2024). Sonographic predictors of antepartum bleeding in placenta previa. Am. J. Perinatol..

[30] Saitoh M., Ishihara K., Sekiya T., Araki T. (2002). Anticipation of uterine bleeding in placenta previa based on vaginal sonographic evaluation. Gynecol. Obstet. Investig..

[31] Nieto-Calvache A.J., Aryananda R.A., Palacios-Jaraquemada J.M., Cininta N., Grace A., Benavides-Calvache J.P., Campos C.I., Messa-Bryon A., Vallecilla L., Sarria D. (2024). One-step conservative surgery vs. hysterectomy for placenta accreta spectrum. A feasibility randomized controlled trial. Am. J. Obstet. Gynecol. MFM.

[32] Munoz J.L., Ramsey P.S., Greebon L.J., Salazar E., McCann G.A., Byrne J.J. (2024). Risk factors of massive blood transfusion (mtp) in cesarean hysterectomy for placenta accreta spectrum. Eur. J. Obstet. Gynecol. Reprod. Biol..

